# Supplementary material for: The Unfolded Protein Response Sensor IRE1 Regulates Activation of In Vitro Differentiated Type 1 Conventional DCs with Viral Stimuli
Source: Int J Mol Sci. 2023 Jun 16;24(12):10205. doi: 10.3390/ijms241210205 (PMC10299223; doi:10.3390/ijms241210205)
Supplement: Supplementary file 1 [file ijms-24-10205-s001.zip › ijms-2416035-supplementary.pdf]

Supplementary Table S1: Antibodies used in this study

ANTIBODY	FLUOROPHORE	CONCENTRATION	CLONE	BRAND
CD11b	FITC	1/400	M1/70	Biolegend
CD11c	APC	1/800	N418	eBioscience
CD11c	BV711	1/800	N418	Biologend
CD11c	PE	1/800	N418	Biolegend
CD16/32 (FcBlock)	Purified	1/400	93	Biolegend
CD172a (SIRPα)	Biotin	1/100	P84	Biolegend
CD172a (SIRPα)	PE-Cy7	1/400	P84	Biolegend
CD172a (SIRPα)	PerCP-Cy5.5	1/400	P84	Biolegend
CD274 (PD-L1)	BV711	1/200	MIH5	BD
CD274 (PD-L1)	PE	1/200	MIH5	Invitrogen
CD40	APC	1/200	3/23	Biolegend
CD44	FITC	1/1600	IM7	Biolegend
CD45R/B220	Biotin	1/100	RA3-6B2	Biolegend
CD80	BUV395	1/200	16-10A1	BD
CD86	PE-Cy7	1/400	GL-1	Biolegend
H2Kb	FITC	1/800	AF6-88.5	Biolegend
IL-12/IL23 p40	eFluor660	1/200	C17.8	eBioscience
IL-6	PE	1/200	MP5-20F3	Biolegend
Streptavidin	PE-Cy7	1/100	-	Biolegend
TNFa	APC	1/200	MP6-XT22	Biolegend
XCR1	BV650	1/400	ZET	Biolegend
XCR1	PE	1/400	ZET	Biolegend

Supplementary Table S2: qPCR primers used in this study

GENE	SEQUENCE	REFERENCE
Erdj4 F	CCCCAGTGTCAAACTGTACCAG	(Lee et al., 2008)
Erdj4 R	AGCGTTTCCAATTTTCCATAAATT	(Lee et al., 2008)
Bip F	ATGAGGCTGTAGCCTATGGTG	Roche Universal Probe Library
Bip R	GGGGACAAACATCAAGCAG	Roche Universal Probe Library

IRE1exón19-20 F	TGCTGAAACACCCCTTCTTC	Roche Universal Probe Library
IRE1exón19-20 R	GCCTCCTTTTCTATTCGGTCA	Roche Universal Probe Library
Chop F	CCACCACACCTGAAAGCAG	Roche Universal Probe Library
Chop R	TCCTGCAGATCCTCATACCAG	Roche Universal Probe Library
Herpud F	AGCAGCCGGACAACTCTAAT	(Wu et al., 2007)
Herpud R	CTTGGAAAGTCTGCTGGACA	(Wu et al., 2007)
Bloc1s1 F	CACCCAGCCAGACTCGAC	(Osorio et al., 2014)
Bloc1s1 R	GCAGCGATAGCTTCTCTCCTC	(Osorio et al., 2014)
l27 F	GCCAAGCGATCCAAGATCAA	Roche Universal Probe Library
l27 R	GCTGGGTCCCTGAACACATC	Roche Universal Probe Library
Il-12a F	GTGAAGACGGCCAGAGAAA	(Satpathy et al., 2013)
Il-12a R	GGTCCCGTGTGATGTCTTC	(Satpathy et al., 2013)
Ifna4 F	CTGCTACTTGGAATGCAACTC	(Fink et al., 2017)
Ifna4 R	CAGTCTTGCCAGCAAGTTGG	(Fink et al., 2017)
Ifnb1 F	GCACTGGGTGGAATGAGACTATTG	(Fink et al., 2017)
Ifnb1 R	TTCTGAGGCATCAACTGACAGGTC	(Fink et al., 2017)
Xbp1exón2 F	CAGCAAGTGGTGGATTTGG	(Tavernier et al., 2017)
Xbp1exón2 R	CGTGAGTTTTCTCCCGTAAAAG	(Tavernier et al., 2017)

Supplementary Figure Legends

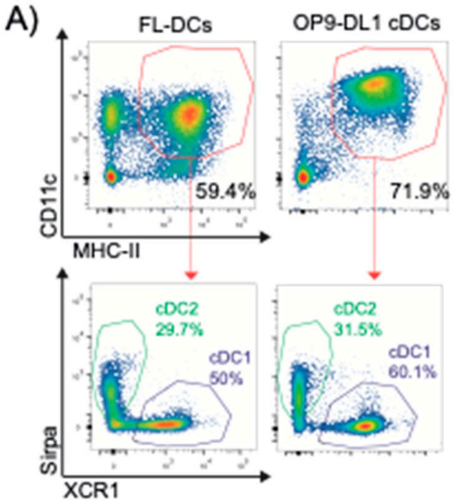

Supplementary Figure S1. Differentiation of cDC1 subpopulation in FL-DCs and OP9-DL1 cDCs cell cultures. A) Comparison between conventional DC cell cultures (FL-DCs) and OP9-DL1 cDCs to evaluate the differentiation into the cDC1 subpopulation. Dot plots depict one representative out of three independent experiments.

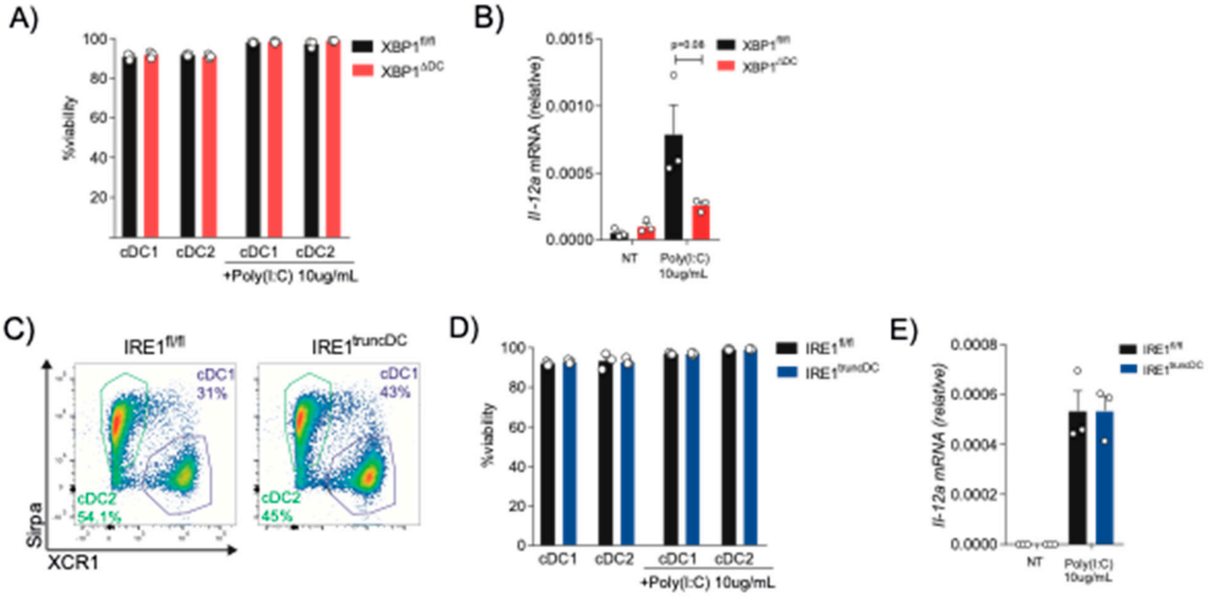

Supplementary Figure S2. Viability of OP9-DL1 cDCs cultures. XBP1ΔDC(A) and (D) IRE1^{truncDC} OP9-DL1 cDCs were cultivated with viral agonist Poly(I:C) to evaluate cell survival. Il12a expression was measured by qPCR in XBP1ΔDC(B) and (E) IRE1truncDC OP9-DL1 cDC1s stimulated with Poly(I:C) 10 µg/mL for 4 h. C) OP9-DL1cDCs cultures from IRE1^{truncDC} and control mice.
